# Mental health and lifestyle in mental health nurses: a cross-sectional, nation-wide study from Uganda during COVID-19 times

**DOI:** 10.11604/pamj.2022.42.210.33928

**Published:** 2022-07-15

**Authors:** Davy Vancampfort, James Mugisha

**Affiliations:** 1 Department of Rehabilitation Sciences, Faculty of Movement and Rehabilitation Sciences, Katholieke Universiteit (KU) Leuven, Leuven, Belgium,; 2 Department of Sociology and Social Administration, Faculty of Arts and Social Sciences, Kyambogo University, Kampala, Uganda

**Keywords:** Physical activity, nursing, sleep, stress, trauma

## Abstract

**Introduction:**

mental health nurses (MHNs) work in potentially high-stress settings, in particular in low-income countries during the COVID-19 pandemic the risk might be high. This multi-centre, cross-sectional study explored the prevalence of psychological distress and post-traumatic stress disorder (PTSD) symptoms among Ugandan MHNs and investigated associations between these mental health outcomes and lifestyle factors.

**Methods:**

in this cross-sectional study, participants completed the Kessler-6 (K-6), PTSD checklist for DSM-5 (PCL-5), simple physical activity questionnaire (SIMPAQ), physical activity (PA) vital sign (PAVS), Pittsburgh sleep quality index (PSQI, and alcohol use disorder identification test-concise (AUDIT-C). Spearman Rho correlations and Mann Whitney U tests were applied.

**Results:**

of 108 included MHNs (age =34.8±10.0 years; 55.6% female) 92.6% had psychological distress (K-6≥13), 44.4% elevated PTSD symptoms (PCL-%≥41), 74.1% was physically inactive (less than 150min/week on PAVS), 75.9% reported poor sleep quality (PSQI>-5) and 24.4% harmful drinking (AUDIT-C≥3 for women and -≥4 for men). SIMPAQ exercise correlated with K-6 (rho =-0.36, P<0.001) and PCL-5 (rho=-0.24, P=0.013), SIMPAQ walking with PCL-5 (rho =-0.31, P<0.001). Mental health nurses meeting the PA guidelines reported lower PCL-5 scores than those who did not (P<0.005).

**Conclusion:**

in Uganda, the mental health burden is high during the COVID-19 pandemic among MHNs and associated with an unhealthy lifestyle. The effectiveness and efficacy of resilience programs for MHNs focusing on unhealthy lifestyle patterns should be explored.

## Introduction

Mental health nurses frequently face emotional adversity and work-related crises including exposure to violence, which can negatively impact their well-being and might result in psychological distress and even post-traumatic stress disorder (PTSD) symptoms [1,2]. An insufficient number of staff and consequently a high workload, time pressure, and role ambiguity might compound the mental burden among mental health nurses even more [3,4]. Psychological distress and symptoms of PTSD can affect the capacity for therapeutic work, professional relationships, and overall work performance [5]. While pre-COVID-19 rates already indicated a prevalence of 5 to 10% for severe depression, anxiety, or stress among mental health nurses from high-income countries [6], this figure rose up to 30% following the first COVID-19 wave [7-9]. Recent reports highlighted challenges in providing care and treatment while wearing personal protective equipment and maintaining social distancing with patients who may not have the capacity to understand guidelines, and within environments that are often unsuitable and unadaptable as the most important reasons [7,8]. To date, data on mental health nurses´ psychological distress and PTSD levels from low-income countries such as Uganda were lacking pre-COVID-19. One might expect that the prevalence was already very high due to the fact that the mental health care system in low-income countries such as Uganda was even before COVID-19 under severe strain due to a lack of human resources, poor working conditions, and poor management [10]. A recent study among Ugandan health care workers following the first COVID-19 wave and including doctors, clinical officers, nurses, midwives, radiographers, but also cleaners, drivers, administrators, laboratory personnel, and support staff working in the regional referral hospitals indicated that 44% experienced psychological distress [11].

The most common concerns reported included fear of COVID-19 infection at the workplace (81%), stigma from colleagues (79%), lack of workplace support (63%), and inadequate availability of personal protective equipment (PPE) (56%) [11]. Data focusing on psychological distress and PTSD symptoms among mental health nurses working in general hospitals are however lacking. In order to address the detrimental impacts of workplace-related psychological distress and PTSD symptoms among mental health nurses, there has been very recently growing attention in high-income countries such as Australia on supporting resilience [5,6,12-14], defined as a process of recovery following adverse events, which involves cognitive, affective, and behavioral self-regulatory responses that support positive adaptation and restoration of psychological well-being and functioning [15]. Existing resilience programs currently focus however mainly on collegial peer group interaction and cognitive-behavioral strategies such as managing negative self-talk and promoting positive self-talk [13]. Future research could also explore the role of supporting a healthy and active lifestyle as part of a resilience program. However, before the effectiveness and efficacy of lifestyle programs in order to reduce psychological distress and PTSD symptoms among mental health nurses should be explored, it is of added value to investigate, as a first step, cross-sectional associations between psychological distress and PTSD symptoms and lifestyle factors in this population at risk. To the best of our knowledge, such data are currently lacking in the international literature. In order to fill the current gaps in the literature, this cross-sectional study aimed: a) to explore levels of psychological distress and PTSD symptoms among Ugandan mental health nurses working in the regional referral hospitals during the COVID-19 pandemic, and b) to investigate associations between psychological distress and PTSD symptom levels and sedentary levels, physical activity levels, sleep quality, and other lifestyle factors such as smoking and harmful drinking. We hypothesize that high levels of psychological distress and PTSD symptoms among Ugandan mental health nurses are associated with being more sedentary and physically inactive, poor sleep quality, and the presence of unhealthy lifestyle factors such as smoking and harmful drinking.

## Methods

**Study design:** we conducted a cross-sectional study exploring the prevalence of psychological distress and post-traumatic stress disorder symptoms among Ugandan mental health nurses and investigated associations between these mental health outcomes and lifestyle factors.

**Study setting:** data were collected in 8 regional referral hospitals across Uganda.

**Study population:** all nurses working in the mental health wards of the included regional referral hospitals were invited to complete the questionnaires on one of two days the research team visited the regional referral hospitals.

**Study sampling:** data were collected in a random selection of 8 of the 13 regional referral hospitals across Uganda. We used Research Randomizer. All regional referral hospitals were given a number. Thirteen sets of numbers were created, and all centers were at random allocated to 0 (not included) or 1 (included). This way, 7 regional referral hospitals were at random given a number 1 and selected. In a second round, for the 6 remaining regional referral hospitals, a new set of numbers was created leaving 2 potential regional referral hospitals as we're given a number 1. In a final round, 1 of the 2 regional referral hospitals was randomly selected, giving a total of 8 regional referral hospitals. All nurses working in the mental health wards of the 8 selected regional referral hospitals were invited on one of two days the research team visited the regional referral hospitals.

**Study variables:** all participants were requested to complete the Kessler - 6 (Kessler *et al*., 2002), PTSD checklist for the DSM-5 (Blevins *et al*., 2015), simple physical activity questionnaire [16], physical activity vital sign [17], Pittsburgh sleep quality index [18], and alcohol use disorder identification test-concise [19], and were asked about their smoking habits.

**Kessler-6 (K-6):** the K-6 (Kessler *et al*., 2002) is a self-report assessment tool that measures psychological distress in the past 30 days using 6 short items on a 5-point likert scale ranging from 1 (all the time) to 5 (none of the time). The K-6 is also used to identify individuals with increased psychological distress drawing from depressive- and anxiety-related symptoms, with a cut-off of 13 indicating severe psychological distress (Kessler *et al*., 2002). The K-6 is a valid instrument that is moderate-to strongly correlated (r=0.65) to other golden-standard assessments Kessler *et al*., 2003.

**Post-traumatic stress disorder checklist for the DSM-5 (PCL-5):** the PCL-5 is a self-report assessment tool that measures symptoms of post-traumatic stress according to the DSM-5 (association, 2013) during the past month with the aim of monitoring symptom change, screening individuals for PTSD, or making a provisional PTSD diagnosis (Blevins *et al*., 2015). The PCL-5 contains 20 items that are scored on a 5-point likert scale, ranging from 0 (not at all) to 4 (extremely). This tool provides an indication for PTSD and a severity score (0-80) with higher scores indicating higher severity. A cut-off score of 41 is recommended for providing a provisional diagnosis of PTSD in first responders (Morrison *et al*., 2021). The PCL-5 has good psychometric properties and is a sound measure of DSM-5 PTSD symptoms (Blevins *et al*., 2015). It has a strong internal consistency (α=0.94), test-retest reliability (r=0.82), and validity (Blevins *et al*., 2015).

**Simple physical activity questionnaire (SIMPAQ):** the SIMPAQ [16] assesses physical activity and sedentary levels among populations at high risk for physical inactivity. More in detail, it estimates time spent in bed (min/day), time spent sedentary during waking hours (min/day), time spent walking (min/day), time spent in structured exercise (min/day), and time spent in incidental or non-structured physical activity (min/day) during the past week. The sum of the hours recorded in the SIMPAQ items should add to approximately 24-hours, providing interviewers with an opportunity to clarify with participants if significant under or over-reporting has occurred (e.g, a total of <18 hours or >30 hours accounted for). In this study, we focus on the time spent sedentary, time spent walking, time spent in structured exercise, and time spent in incidental or non-structured physical activity. Previous research in Uganda demonstrated the questionnaire is reliable [20] while the validity has been demonstrated in a 23-country validation study [16].

**Physical activity vital sign (PAVS):** the PAVS [17] comprises two simple questions. The first question is: “On average, how many days per week do you engage in moderate to vigorous physical activity like a brisk walk?” It was explained to the health care professionals that this means that due to being active their heart rate increased, and they breathed more deeply and faster than normal, with some maybe even experiencing sweating. The second question is: “On those days, how many minutes do you engage on average in physical activity at this level?” Next, the two responses were multiplied to calculate the minutes per week of self-reported moderate to vigorous physical activity. The total score was used as a dichotomous outcome in order to investigate whether nurses were achieving the internationally recommended target of 150 minutes per week of moderate to vigorous physical activity. The PAVS has been validated [21] and used before in Ugandan physical activity studies in mental health nurses [22,23].

**Pittsburgh sleep quality index (PSQI):** the 19-item PSQI [18] assesses subjective sleep quality in the last month. Items are categorized into seven components: subjective sleep quality, sleep latency, sleep duration, habitual sleep efficiency, sleep disturbances, sleep medication, and daytime dysfunction. The possible score range for each component is 0 (no difficulty) to 3 (severe difficulty). The seven component scores are summed to produce a global score; higher scores represent poorer subjective sleep quality. The total score was used as a continuous measure but also as a dichotomous outcome in order to explore the differences between poor and good sleepers. Previous research demonstrated that a global PSQI score >5 yields a diagnostic sensitivity of 89% and specificity of 86.5% (κ=0.75, p ≤0.001) in distinguishing “good” from “poor” sleepers. Therefore, “good sleep” can be defined as a global PSQI score of 0 to 5 and “poor sleep” as a global PSQI scores of 6 to 12 [18].

**Harmful drinking:** alcohol use disorder identification test-concise (AUDIT - C). Alcohol use was measured with the AUDIT-C (AUDIT questions 1-3) [19]. The questions assessed frequency of drinking, the typical number of drinks consumed on a drinking day, and frequency of binge-drinking during the past 12 months. Responses to each item were scored from 0 to 4. A score on the AUDIT-C of 3 or greater is considered positive in women, 4 or greater for men. The AUDIT-C is a sensitive tool to assess harmful drinking in Uganda [24].

**Smoking:** participants were asked whether they smoked or not, and if so, how many cigarettes they smoke per day on average.

### Study procedure

**Power analysis:** first, a power analysis using G*power (3.1.9.7) [25] for simple correlation analyses was executed. This power analysis demonstrated that a sample size of 84 participants should be sufficient in order to detect a small effect (r= 0.30) [26] with a statistical power of 80% and a significance level of 0.05. This power is valid for the correlation analyses. Group comparisons were carried out on an exploratory basis.

**Data collection:** data were collected between January 2021- March 2021 following the first wave of COVID-19 and between September and November 2021 following the second wave of COVID-19. Patients, who were willing to participate, completed the questionnaire before a workshop on self-care.

**Statistical analyses:** normality was tested by means of the Shapiro-Wilk test and data were, except for age, found not to be normally distributed. All data except age are presented as medians and interquartile ranges. Missing data were replaced by the median of the nearby points. In case, Associations between lifestyle measures and mental health outcomes were investigated with Spearman Rho correlations with non-parametric 95% boot strap confidence intervals (CIs). To interpret the strength of correlation, we used Cohen cut-off points of 0.10, 0.30, and 0.50: with r < 0.30 considered as a small correlation, 0.30 ≤ r < 0.50 as a medium correlation, and r 0.50 as a large correlation [26]. Differences in mental health outcomes between those who did and did not meet the minimum physical activity recommendation of at least 150 min of moderate to vigorous physical activity per week, between poor and good sleepers, between hazardous and non-hazardous drinkers and smokers and non-smokers were investigated using Mann Whitney U - tests.Differences in the number of participants scoring above the cut-off for unhealthy lifestyle or mental health outcomes between both waves were explored with Fisher exact tests. Significance levels were set at P<0.05 for all analyses. The statistical analyses were performed with SPSS version 28.

**Ethical considerations:** the study was conducted in accordance with the Declaration of Helsinki [27]. The study procedure was approved by the Mild may Uganda Research Ethics Committee (MUREC) (0405/2020). All participants gave their written informed consent. No financial compensation was provided, but all mental health wards received a 4-hour self-care workshop by the research team.

## Results

**Characteristics of participants:** none of the eligible mental health nurses working on the wards during the two testing days did refuse to complete the questionnaires (n=110). However, two files were excluded as the responses on the questionnaires had zero variance (i.e, participants gave the same response for all questions) (response rate = 98.2%). In total, 108 participants (age= 34.8 ± 10.0 years; 55.6% female, wave 1= 60, wave 2= 48) were included in the current study, indicating the study has sufficient power. Of these, exactly 100 or 92.6% scored above the cut-off score for psychological distress (K-6 ≥ 13; median =24.0; interquartile range =9.0) and 48 or 44.4% above the cut-off score for PTSD (PCL-5 ≥ 31; median =28.5; IQR =35.0). There was no difference in the number of participants scoring above the K-6 (P=0.28) and PCL-5 (P=0.44) cut-offs between the first and second COVID-19 wave. Eighty (74.1%) participants did not meet the physical activity guidelines, 82 or 75.9% reported poor sleep quality and 26 or 24.4% scored above the cut-off for harmful drinking (AUDIT-C ≥ 3 in women, ≥4 in men). Medians and IQRs were 17.0 (2.0) hours per day, 10.0 (23.2) minutes per day, 7.8 (15.0) minutes per day, 2.0 (60.0) minutes per day, 1.0 (0.0), and 0.0 (2.0) for SIMPAQ sedentary, SIMPAQ walking, SIMPAQ exercise, SIMPAQ incidental physical activity, PSQI and AUDIT-C respectively. There was only one male smoker who smoked two cigarettes per day. There was no difference between the first and second COVID-19 wave in the number of those who did meet physical activity guidelines (P=0.83), reported poor sleep quality (P=0.99), and those scoring above the cut-off for harmful drinking (P=0.99). There were no differences in PCL-5, K-6, SIMPAQ, PSQI and AUDIT-C scores between male and female nurses. There were also no significant associations between age and the continuous PCL-5, K-6, SIMPAQ, PSQI and AUDIT-C scores, nor age differences in those who did meet versus did not meet physical activity guidelines, poor sleep quality and those scoring above the cut-off for harmful drinking.

**Associations between continuous lifestyle and mental health outcomes in Ugandan mental health nurses:** as can be noticed in Table 1, small, significant Spearman Rho correlations were found between the PCL-5 total score and the SIMPAQ walking and SIMPAQ exercise scores. Similarly, a small significant correlation was found between the K-6 total score and the SIMPAQ exercise. No other significant correlations were found.

**Table 1:** associations between lifestyle factors and mental health outcomes in 108 Ugandan mental health nurses working in general referral hospitals

	PCL-5			K-6		
	r	95% CI	P	R	95% CI	P
SIMPAQ sedentary (hours/day)	0.15	-0.04 to 0.33	0.12	-0.15	-0.33 to 0.04	0.13
SIMPAQ walking (min/day)	-0.31	-0.47 to -0.13	<0.001*	-0.09	-0.27 to 0.10	0.37
SIMPAQ exercise (min/day)	-0.36	-0.51 to -0.18	<0.001*	-0.24	-0.41 to 0.05	0.013*
SIMPAQ incidental physical activity (min/day)	-0.09	-0.27 to 0.1	0.36	-0.07	-0.25 to 0.12	0.47
PSQI total score	0.11	-0.08 to 0.29	0.28	-0.14	-0.32 to 0.05	0.13
AUDIT-C total score	0.001	-0.19 to 0.19	0.99	-0.04	-0.23 to 0.15	0.70

Spearman Rho correlations; significance level set at P<0.05; Audit- C: alcohol use disorder identification test; concise; K-6, Kessler 6; PSQI: Pittsburgh sleep quality index; PCL-5: PTSD checklist for the DSM-5; SIMPAQ: simple physical activity questionnaire

**In mental health outcomes between subgroups of mental health nurses according to lifestyle factors:**mental health nurses meeting the physical activity guidelines reported lower PCL-5 [15.0 (8.0) versus 33.0 (38.2)], P<0.001) and K-6 [22.0 (7.0) versus 25.0 (8.0), P=0.005] scores than those who did not. There was no significant difference in PCL-5 [29.0 (31.5) versus 16.0 (40.5)], P=0.27) and K-6 [24.0 (9.0) versus 25.0 (6.0), P=0.13] between those who reported a poor sleep quality and those who did not. Similarly, there was no significant difference in PCL-5 [38.0 (33.2) versus 28.0 (35.2)], P=0.82) and K-6 [24.0 (6.5) versus 24.0 (9.2), P=0.51] between those who reported harmful drinking patterns versus those who did not.

## Discussion

The current nationwide study in 8 regional referral hospitals and with a response rate of over 98% demonstrates that the mental health burden in mental health nurses working in a low-income country such as Uganda during the COVID-19 pandemic is very high. Almost 93% of 108 nurses scored above the cut-off for psychological distress based on the K-6 and more than 44% scored above the cut-off for PTSD based on the PCL-5. Our data indicate that resilience programs for mental health nurses, which in recent years have been introduced in high-income countries [13], are urgently needed in low-income countries such as Uganda as well. Our data furthermore demonstrate that lower levels of psychological distress and PTSD symptoms are observed in those who are sufficiently physically active, i.e those mental health nurses who meet the recommendation of the World Health Organization of at least 150 minutes of physical activity per week at, at least, moderate-intensity [28]. Therefore, the current data provide an important rationale to explore the effectiveness and efficacy of the promotion of an active lifestyle as an important component of resilience programs. This is in line with very recent calls for higher-quality research establishing the efficacy and cost-effectiveness of different resilience interventions [29]. Considering the high levels of physical inactivity, i.e 74% did not meet the physical activity recommendation of the World Health Organization [28], such programs might even benefit the majority of mental health nurses. Since associations were found for psychological distress with exercise levels and for PTSD symptoms with exercise and walking levels, such studies should, in particular, investigate whether increasing leisure and transport-related physical activity levels in mental health nurses might reduce the mental health burden.

There is evidence from the general population that increasing leisure-time physical activity and, to a lesser extent, transport-related physical activity improve mental wellbeing and reduce depression and anxiety more so than occupation-related physical activity or incidental physical activity [30,31]. Future studies in low-income countries such as Uganda should also explore the underlying mechanisms for the observed associations in more detail. There is evidence from clinical and non-clinical populations that several neurobiological and psychosocial pathways could clarify a reduction in mental health symptoms when being physically active. For example, neurobiological changes (e.g, increased cerebral blood volume and/or flow) and changes in peripheral biomarkers (e.g, increase in circulating growth factors, and anti-inflammatory markers) have been reported before in those being more physically active [32,33], while from a psychosocial perspective there is evidence that physical activity provides an opportunity for social interaction [34], and increases mastery in the physical domain (e.g, self-perceived fitness, self-esteem, self-efficacy) [35,36]. In contrast to what was hypothesized, and what has been observed recently in Chinese mental health nurses using similar assessment tools [37], we did not find any associations between poor sleep quality and psychological distress. While in the current study, 75.9% reported poor sleep quality and 92.6% psychological stress, among 812 Chinese mental health nurses working in a psychiatric hospital this was 53.1% used the PSQI and 46.9% used the K-10 instead of K-6 respectively. More research is needed to clarify the discrepancy with recent research, although a lack of power in our exploratory group comparisons might be a reason. Finally, the exploratory analyses did also not show significant differences in PCL-5 and K-6 between mental health nurses reporting harmful drinking levels versus those who did not. On the other hand, the high prevalence of harmful drinking, based on the AUDIT-C, in Ugandan mental health nurses is concerning, i.e. almost one in four scored above the cut-off score. With nurses´ health linked to their job satisfaction [38], this might have direct implications for the work environment and the care of patients. There might also be implications for the Ugandan community at large. Health education and health promotion have been identified as key roles in the profession [39]. That nurses have a responsibility as role models for the community is questioned by some [40], but there is little challenge to the presumption that health professionals´ behaviors are an influence on the population [41].

**Limitations:** although the current findings indicate that a focus on the mental health burden and unhealthy lifestyle of mental health nurses in a low-income country such as Uganda is urgently needed, our data should be considered with caution. First, due to the cross-sectional design, the directionality of the relationships remains uncertain. As indicated, intervention studies are needed to disentangle the observed relationships. Second, the mental health nursing staff self-reported their physical activity and sedentary levels, sleep quality, drinking levels, and mental health status, potentially introducing reporting bias into the analysis. Future research should use objective diagnostic interviews and physical activity devices, such as accelerometers-inclinometers

**Funding:** this research was support by the Vlaamse Interuniversitaire Raad (VLIR-UOS) with a South Initiative grant (number SI-2019-01-21).

## Conclusion

Despite the abovementioned limitations, the current data clearly demonstrate that almost all mental health nurses working during the COVID-19 pandemic in the regional hospitals in Uganda experience psychological distress, and almost half of them experience elevated post-traumatic stress. High levels of psychological distress and post-traumatic stress are associated with an unhealthy lifestyle, with almost three out of four mental health nurses being insufficiently physically active and reporting poor sleep, and one out of four reporting harmful drinking levels. From a policy and management perspective, mental health care settings should put strategies in place to reduce the high mental health burden of its nursing staff. Resilience programs for mental health nurses focusing on the importance of an active lifestyle should be investigated.

### What is known about this topic

- *While pre-COVID-19 rates already indicated a prevalence of 5 to 10% for severe depression and anxiety among mental health nurses from high-income countries, COVID-19 worsened the mental health burden in this population even more;*
- *Existing resilience programs for mental health nurses focus on collegial peer group interaction and cognitive-behavioural strategies such as managing negative self-talk and promoting positive self-talk but not on a healthy and active lifestyle*.

### What this study adds

- *Almost all (93%) mental health nurses in Uganda report high psychological distress and almost half (45%) high levels of posttraumatic stress during the COVID-19 pandemic;*
- *With about three fourth not meeting the international physical activity guidelines and reporting poor sleep quality and one fourth scoring above the cut-off for harmful drinking, mental health nurses in Uganda live a very unhealthy life*.
